# Development of a Multiplex and Cost-Effective Genotype Test toward More Personalized Medicine for the Antiplatelet Drug Clopidogrel

**DOI:** 10.3390/ijms15057699

**Published:** 2014-05-05

**Authors:** Hye-Eun Jeong, Su-Jun Lee, Eun-Young Cha, Eun-Young Kim, Ho-Sook Kim, Young Hwan Song, Jae-Gook Shin

**Affiliations:** 1 Department of Pharmacology and Pharmacogenomics Research Center, Inje University, Busan 614-812, Korea; E-Mails: milkfrance@hanmail.net (H.-E.J.); 2sujun@inje.ac.kr (S.-J.L.); televy98@hanmail.net (E.-Y.C.); eykim@inje.ac.kr (E.-Y.K.); hosuegi@gmail.com (H.-S.K.); 2 Department of Microbiology, Pukyoung National University, Busan 608-737, Korea; E-Mail: yhsong@gmail.com; 3 Department of Clinical Pharmacology, Inje University College of Medicine, Inje University Busan Paik Hospital, Inje University, Busan 614-812, Korea

**Keywords:** clopidogrel, genotypes, *CYP2C19*, *P2Y12*, SNaPshot, pharmacogenetics

## Abstract

There has been a wide range of inter-individual variations in platelet responses to clopidogrel. The variations in response to clopidogrel can be driven by genetic polymorphisms involved in the pathway of absorption, distribution, metabolism, excretion, and the target receptor P2Y12. A set of genetic variants known for causing variations in clopidogrel responses was selected, which included CYP2C19*2, *3, *17, CYP2B6*4, *6, *9, CYP3A4*18, CYP3A5*3, *MDR1 2677G* > *T*/*A*, *3435C* > *T*, and *P2Y12 H2* (*742T* > *C*). The simultaneous detection of these 10 variants was developed by using a multiplex PCR and single-base extension (MSSE) methodology. The newly developed genotyping test was confirmed by direct DNA sequencing in the representative positive control samples and validated in an extended set of 100 healthy Korean subjects. Genotyping results from the developed MSSE exhibited a perfect concordance with the direct DNA sequencing data and all of variants tested in 100 healthy Korean subjects were in agreement with Hardy-Weinberg equilibrium (*p* > 0.05). The present molecular diagnostic studies provide an accurate, convenient, and fast genotyping method for the detection of multiple variants. This would be helpful for researchers, as well as clinicians, to use genetic information toward more personalized medicine of clopidogrel and other antiplatelet drugs in the future.

## 1. Introduction

Clopidogrel is a member of the thienopyridine class of adenosine diphosphate (ADP) receptor inhibitor, which inhibits platelet aggregation by irreversibly binding to *P2Y12* receptor on platelet membrane [1]. The response to clopidogrel differs widely from subject to subject, and about 25% of patients treated with standard clopidogrel dose exhibit insufficient inhibition of ADP-induced platelet aggregation [2]. Although the U.S. food and drug administration (FDA), in March 2010, issued a black box warning that individuals with low levels of CYP2C19 expression may not get the full effect of clopidogrel, the underlying mechanism responsible for the clopidogrel non-responsiveness and/or adverse effects are complicated and unclear. Multiple studies have indicated that functional variants of cytochrome P450s were associated with different metabolism of clopidogrel, which include CYP3A4/5, 2C19, and 2B6 [2–4]. In addition, the efflux transporter P-glycoprotein 1, also known as ATP-binding cassette sub-family B1 (*ABCB1*), has been reported to affect clopidogrel bioavailability [5]. For example, individuals carrying *ABCB1 3435 TT* genotypes have exhibited reduced platelet inhibition with increased risk of recurrent ischemic events during anti-platelet drug treatment [4]. In patients with acute coronary syndromes who have undergone percutaneous intervention, nearly half of the individuals having major adverse cardiovascular events were found to carry a genotype associated with increased risk alleles of *ABCB1* and *CYP2C19* [5], suggesting that the improved prediction of cardiovascular events could be possible when combined with both *ABCB1* and *CYP2C19* genotypes than the application of individual gene alone. In addition, genetic polymorphisms in the *P2Y12* gene have been suggested to contribute to inter-individual variations in clopidogrel response in patients [6]. Thus, genetic polymorphisms linked to functional variation in genes related to clopidogrel pharmacokinetics and pharmacodynamics are expected to alter active metabolite formation and its local concentration, which may lead to inter-individual variations in clopidogrel responses. The combined genotype analysis covering multiple genes could be helpful in understanding genetic influences on the drug response variations. For the development of genotyping assays, we there by selected 10 important SNPs from multiple genes based on their clinical implications and relatively high frequency in Asian population [7–10].

There are various genotyping methods available, such as High Resolution Melting (HRM), Pyrosequencing, and Taqman assay. All of three representative methods are convenient to use, however, they can detect only limited number of genetic markers, mostly up to 1–3 single nucleotide polymorphisms (SNPs), in a reaction. Therefore, the objective of our study was to develop a simultaneous detection method for a set of genetic markers by using a multiplex SNaPshot single-base extension (MSSE) in a single reaction.

## 2. Results and Discussion

### 2.1. Results

The goal of the present study was to develop an accurate, fast and cost-effective genotyping method using MSSE strategy for the detection of a set of multiple variants known for associated with variable clopidogrel responses. To obtain the expected high-throughput of the multiplex PCR for the multiple targets, the PCR was optimized by adjusting several different factors, which include primer specificity, primer annealing temperature, prevention from secondary-structure formation by primer itself, and primer—primer self-complementary structures. Bioinformatic software, PSQ assay design (Biotage-Qiagen, Valencia, CA, USA), was used to have the low probability of secondary structures of hairpin and dimer formation in all the ten pairs of primers. As expected, PCR conditions were optimized and exhibited the specific amplification of the target genes (Table 1).

Since there is high homology of DNA sequence among the same subfamily of genes, specificity of the all PCR products were verified by direct DNA sequencing. After the two multiplex PCRs, the detection of 10 variants in six genes was performed by single-base extension (SBE) and achieved a specific separation of all different alleles in a single reaction. In particular, SBE requires optimized concentration of each probe to avoid unspecific products and to produce similar levels of fluorescent intensities with the neighbors. Different amounts of SBE sequencing probes were determined as shown in Table 2 (0.01–0.12 nM).

**Figure 1.**
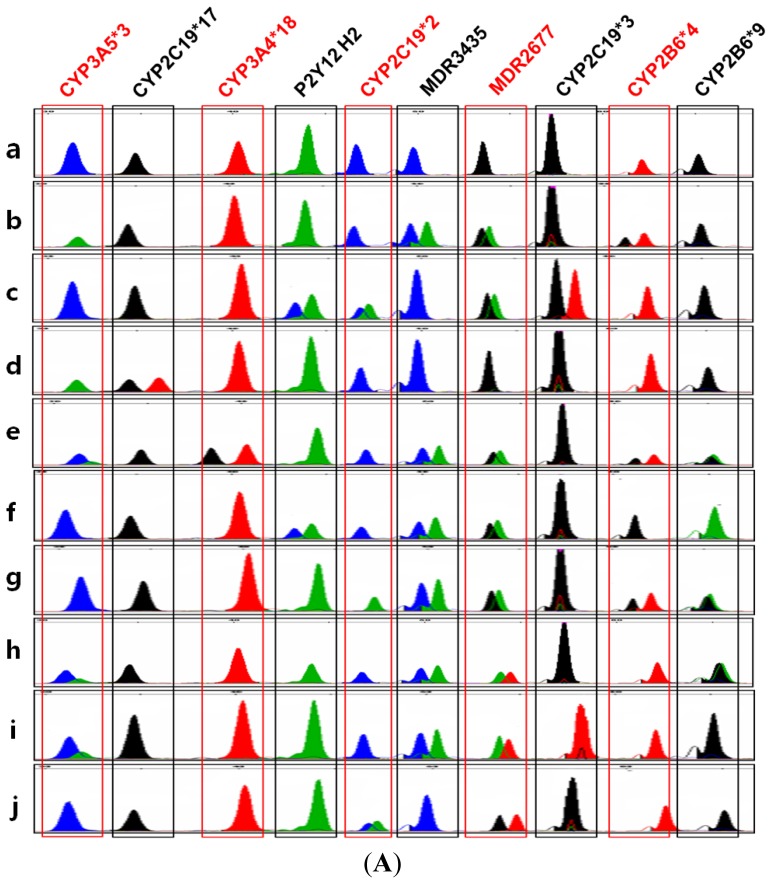

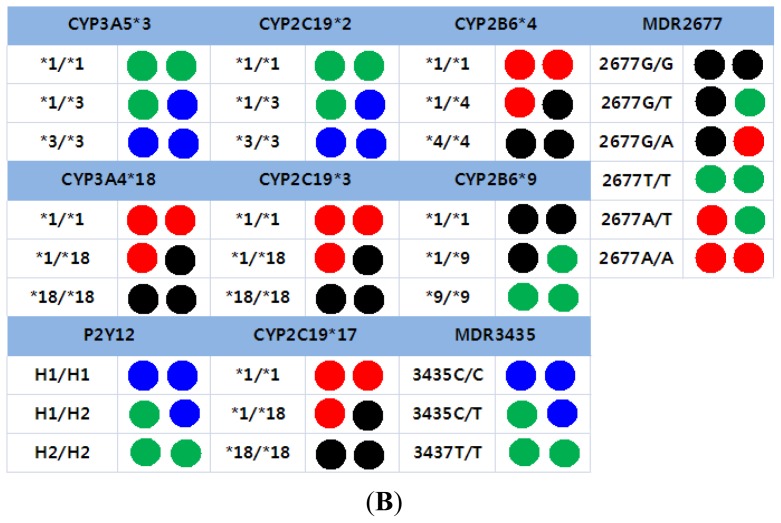
(**A**) Representatives of electropherograms obtained from multiplex single-base extension. The detected variant alleles are shown above the peaks (Green: A, Red: T, Blue: G, and Black: C). Genotypes of ten samples are illustrated as representative results. (**a**) CYP2B6 *1/*1, CYP2C19 *1/*1, CYP3A4 *1/*1, CYP3A5 *3/*3, *MDR1 2677 G*/*G*, *MDR1 3435 C*/*C*, and *P2Y12 H1/H1;* (**b**) CYP2B6 *1/*4, CYP2C19 *1/*1, CYP3A4 *1/*1, CYP3A5 *1/*1, *MDR1 2677 G*/*T*, *MDR1 3435C*/*T*, and *P2Y12 H1*/*H1;* (**c**) CYP2B6 *1/*1, CYP2C19 *2/*3, CYP3A4 *1/*1, CYP3A5 *3/*3, *MDR1 2677G*/*T*, *MDR1 3435C*/*C*, and *P2Y12 H1*/*H2;* (**d**) CYP2B6 *1/*1, CYP2C19 *1/*17, CYP3A4 *1/*1, CYP3A5 *1/*1, *MDR1 2677 G*/*G*, *MDR1 3435 C*/*C*, and *P2Y12 H1/H1;* (**e**) CYP2B6 *1/*6, CYP2C19 *1/*1, CYP3A4 *1/*18, CYP3A5 *1/*3, *MDR1 2677 G/T*, *MDR1 3435 C*/*T*, and *P2Y12 H1*/*H1;* (**f**) CYP2B6 *6/*6, CYP2C19 *1/*2, CYP3A4 *1/*1, CYP3A5 *3/*3, *MDR1 2677 G*/*T*, *MDR1 3435 C*/*T*, and *P2Y12 H1/H2* (**g**) CYP2B6 *1/*6, CYP2C19 *2/*2, CYP3A4 *1/*1, CYP3A5 *3/*3, *MDR1 2677 G*/*T*, *MDR1 3435 C*/*T*, *P2Y12 H1*/*H1;* (**h**) CYP2B6 *1/*9, CYP2C19 *1/*1, CYP3A4 *1/*1, CYP3A5 *1/*3, *MDR1 2677 A*/*T*, *MDR1 3435C*/*T*, and *P2Y12 H1/H1;* (**i**) CYP2B6 *1/*1, CYP2C19 *3/*3, CYP3A4 *1/*1, CYP3A5 *1/*3, *MDR1 2677 A*/*T*, *MDR1 3435 C*/*T*, and *P2Y12 H1/H1;* (**j**) CYP2B6 *1/*1, CYP2C19 *1/*2, CYP3A4 *1/*1, CYP3A5 *3/*3, *MDR1 2677 G/A*, *MDR1 3435 C*/*C*, and *P2Y12 H1*/*H1;* (**B**) A Schematic of genotype calling for each variant allele and genotype.

The electrograms for CYP2B6*4, *6, *9, CYP2C19*2, *3, *17, CYP3A4*18, CYP3A5*3, *MDR1 2677G>T/A*, *MDR1 3435C>T*, and *P2Y12 H2* are shown in Figure 1A.

The developed MSSE was applied to an extended set of 100 healthy Korean subjects. Frequencies of CYP2C19*2, CYP2C19*3, CYP2C19*17, CYP3A4*18, CYP3A5*3, CYP2B6*4, CYP2B6*6, CYP2B6*9, *P2Y12 H2*, *MDR1 2677G>T/A*, and *MDR1 3435C>T* are indicated in Table 3. To test inter-assay variations, MSSE genotyping of 100 different individuals was performed twice and no differences were found between two tests. Frequencies of variants were in agreement with Hardy–Weinberg equilibrium in study population (*p* > 0.05). Although the present study investigated the allele frequencies in healthy normal population for the validation of detection method, the frequencies in clinical patients may be different from the healthy control subjects. Direct sequencing results showed 100% concordance with those of the Multiplex Single-Base Extension methods (Table 3), indicating perfect specificity and sensitivity for the new MSSE method for the detection of 10 variants alleles.

### 2.2. Discussion

Polymorphisms leading to functional alterations in genes that modulate clopidogrel pharmacokinetics and pharmacodynamics are expected to alter active metabolite formation and anti-platelet effects, leading to poor clinical outcome or adverse drug reaction. Clopidogrel must be bio-transformed into its active metabolite to exert its antiplatelet effects, which is accomplished by hepatic cytochrome P450 isoenzymes, including CYP2C19, CYP2B6, CYP3A4, and CYP3A5 [17,18]. Particularly, CYP2C19 has received considerable attention due to its major role in the formation of active form of clopidogrel. For example, loss of CYP2C19 function by CYP2C19*2, *3 was reported to be associated with poor clinical outcomes after an acute coronary syndrome, particularly after percutaneous coronary intervention [2,3,10]. The efflux transporter P-glycoprotein *C3435T* has also been shown to affect clopidogrel absorption [19], exhibiting a correlation with platelet reactivity in acute coronary syndrome patients [20]. In multiple studies, *P2Y12* is the pharmacological target of clopidogrel and *P2Y12 T742C* polymorphism has been suggested to be associated with clopidogrel outcome in patients with coronary artery disease after coronary stenting [3,20–22].

Drug response is affected by genetic and environmental factors. Depending on the magnitude of severity of genetic mutation in the drug response pathway or variable environmental impacts, phenotypic consequences caused by single gene mutation could be masked or exaggerated. Therefore, genotyping method detecting multiple variations together rather than a single gene variant detection would be more robust for understanding the relationship between genotypes and phenotypes, in particular, in complex diseases, such as cardiovascular diseases. Two drugs, ticagrelor and prasugrel, are kinetically independent from *CYP2C19* genotype and they may have an advantage over clopidogrel in this sense. Therefore, the present genotyping method for the clopidogrel responsiveness may be useful in decision making for drug selection, at least in part.

The translation of pharmacogenomics to the clinical practice has been a difficult task. Multiple reports for clinical practice guidelines to help clinicians in selecting the best management strategy for an individual patient have been presented and updated in several institutions, which include the Clinical Pharmacogenetics Implementation Consortium (CPIC) guidelines for *CYP2C19* and clopidogrel therapy [23], the American College of Cardiology Foundation/American Heart Association (ACCF/AHA) guideline for the management of heart failure [24], and the Royal Dutch Association for the Advancement of Pharmacy (KNMP)—Pharmacogenetics Working Group (PWG) guidelines for the development of pharmacogenetics-based therapeutic dose recommendations [25].

One of the benefits of MSSE methodology, in addition to the detection of multiple variants simultaneously, is its cost-effectiveness. When the cost of genotyping using MSSE methodology was estimated by using the commercial source of reagents in our study, the genotyping cost for a set of 10 SNPs was estimated to be about $10. Cost-effectiveness of genotype-guided antiplatelet therapies was reported in acute coronary syndrome patients, suggesting that genotype-guided personalization may improve the cost-effectiveness of prasugrel and ticagrelor [26]. Comparative analysis of six genotype platforms including a MSSE is presented in Table 4 [27–33].

MSSE method can also be used flexibly, since new alleles can be added to the pre-existent protocol. Flexibility of the SNP selection in the set of genotyping is important for researchers to study genetic effects of new alleles on phenotypic variations in humans. It is generally accepted that once fixed chip-based genotyping platforms is difficult to change with different variants in its system [34]. However, various genotyping approaches may eventually be superseded by the next-generation DNA sequencing with its falling cost of sequencing technologies that can potentially access all form of variations in the target genes. In summary, genetic variants involved in pharmacokinetics and pharmacodynamics of clopidogrel were selected, which include 10 variants from six genes. Genotyping method using MSSE for the detection of these 10 variants was successfully developed. The present molecular diagnostic method for the study of clopidogrel response would facilitate understanding of variations in clopidogrel response and this method may also be extended to the similar agents of drug response in the future.

## 3. Experimental Section

### 3.1. Subjects and DNA Samples

Genomic DNAs used in this study were obtained from 100 healthy volunteers whose genetic materials were deposited in the DNA repository bank at INJE Pharmacogenomics Research Center (Inje University College of Medicine, Busan, Korea). Written informed consent was obtained from all volunteers and the research protocol for the use of human DNA from blood samples was approved by the Institutional Review Board (IRB) of Busan Paik Hospital (Busan, Korea). Genomic DNA was extracted from peripheral blood cells using QIAamp DNA Blood Mini Kit (Qiagen, Chatsworth, CA, USA).

### 3.2. Multiplexing PCR and SNaPshot

For amplifications of the various genes (*CYP2B6*, *CYP2C19*, *CYP3A4*, *CYP3A5*, *MDR1*, *P2Y12*), two multiplexing PCR reactions were performed. One contained CYP2B6*9, CYP2C19*2, CYP2C19*3, CYP2C19*17, CYP3A4*18, CYP3A5*3, and *MDR1 3435C*>*T* at an annealing temperature of 57 °C. The other included CYP2B6*4, *MDR1 2677G>T/A*, and *P2Y12 H2* at an annealing temperature of 63 °C. The amplification primers and PCR condition are described in Table 1. Briefly, PCR was performed using a 9700 Thermal Cycler (PE Applied Biosystems, Foster City, CA, USA) with the following conditions: initial denaturation at 94 °C for 5 min, followed by 35 cycles of 94 °C for 30 s, 57 or 63 °C for 30 s, 72 °C for 30 s, and a final elongation step at 72 °C for 5 min. The pooled PCR products were purified by ExoSAP-IT (USB-Affymetrix, Cleveland, OH, USA) and used as a template to detect 10 polymorphic positions of various genes (Table 2). The primer lengths were designed to avoid overlapping peak signals by spacing mostly five nucleotides (Table 2). Multiplex single-base extension was performed using SNaPshot in accordance with the manufacturer’s protocol (Applied Biosystems, Foster City, CA, USA). Briefly, prepared samples were denatured at 95 °C for 5 min and run on an ABI-Prism 3100 (Applied Biosystems, Foster City, CA, USA) genetic analyzer using a 36-cm capillary array and POP-7 polymer. Turnaround time for the detection of 10 variants was 4 h in the present method. Analyses were performed with GeneMapper software (Applied Biosystems version 3.7, Foster City, CA, USA). The presence of CYP2C19*2, *3, *17, CYP3A4*18, CYP3A5*3, CYP2B6*4, *6, and *9 alleles, and *P2Y12 H2*, *MDR1 2677G>T/A*, and *MDR1 3435C>T* was confirmed by direct DNA sequencing.

### 3.3. Statistical Analysis

Hardy–Weinberg equilibrium was tested for the genotyped SNPs by SNPAlyze software (version 4.1; Dynacom Co., Ltd., Yokohama, Japan) to see genotyping error and population stratification.

## 4. Conclusions

A ten of genetic variants known for causing variations in clopidogrel responses was selected and the simultaneous detection of these ten variants was developed by using a multiplex PCR and single-base extension (MSSE) methodology. The present genotyping method provides an accurate, fast, and cost-effective genotyping method for pharmacogenomic studies of clopidogrel. To the best of our knowledge, the present method is the first that can screen for multi-polymorphisms in multiple genes related to the clopidogrel responsiveness. Application of our method would be useful in estimating the genetic contribution to the variable responses of clopidogrel in Koreans, as well as ethnically related other Asian populations.

## Figures and Tables

**Table 1.** List of primers used in multiplex PCR.

Alleles	Primer Sequence (5′–3′)	Size (bp)	Annealing Temperature (°C)	References
CYP2B6*9	F-GGTCTGCCCATCTATAAAC R-CTGATTCTTCACATGTCTGCG	526	57	[11]
CYP2C19*2	F-AATTACAACCAGAGCTTGGC R-TATCACTTTCCATAAAAGAAG	169	57	[12]
CYP2C19*3	F-CCAATCATTTAGCTTCACCC R-ACTTCAGGGCTTGGTCAATA	262	57	[12]
CYP2C19*17	F-GCCCTTAGCACCAAATTCTCT R-CACCTTTACCATTTAACCCCC	483	57	[13]
CYP3A4*18	F-CACATCAGAATGAAACCACC R-AGAGCCTTCCTACATAGAGTCA	450	57	[14]
CYP3A5*3	F-CATGACTTAGTAGACAGATGA R-TATGTTATGTAATCCATACCCC	423	57	[15]
MDR3435	F-GGGTGGTGTCACAGGAAGAG R-CATGCTCCCAGGCTGTTTAT	113	57	[16]
CYP2B6*4	F-GACAGAAGGATGAGGGAGGAAGATG R-CTCCCTCTGTCTTTCATTCTGTCTTC	640	63	[11]
MDR2677	F-TGTTGTCTGGACAAGCACTGA R-GCATAGTAAGCAGTAGGGAGTAACAA	141	63	[16]
P2Y12 H2	F-TGCTGAAAATTGAAGCCATACTGT R-GCATCTACATCTTGGGAATTTGAA	278	63	-

**Table 2.** List of sequencing probe used in the single-base extension.

Alleles	Location	Primer Sequence (5′–3′)	Concentration (nM)
3A5*3 seqF (P21)	intron03	(T)_3_AGAGCTCTTTTGTCTTTCA	0.12
CYP2C19*17 seqF (P30)	promoter	(T)_0_TTGTGTCTTCTGTTCTCAAAG	0.04
3CYP3A4*18 SeqF (P19)	exon10	(T)_8_CTCCTTTCAGCTCTGTCCGATC	0.02
P2Y12 H2 seqR (P38)	intron02	(T)_12_CTACATCTTGGGAATTTGAAATGAC	0.02
CYP2C19*2 seqF (P55)	exon05	(T)_20_CACTATCATTGATTATTTCCC	0.08
MDR1 3435 seqR (P39)	exon26	(T)_24_GCCTCCTTTGCTGCCCTCAC	0.01
MDR1 2677 seqR (P45)	exon21	(T)_26_AGTTTGACTCACCTTCCCAG	0.03
CYP2C19*3 seqR (P50)	exon04	(T)_28_CAAAAAACTTGGCCTTACCTGGAT	0.04
CYP2B6*4 seqR (P60)	exon05	(T)_35_AGGTAGGTGTCGATGAGGTCC	0.12
CYP2B6*9 seqR (P65)	exon04	(T)_38_GATGATGTTGGCGGTAATGGA	0.12

**Table 3.** Frequency and concordance of detected variants with DNA sequencing in a Korean population (*n* = 100).

SNP	rs Number	Effect	Frequency (%) (95% CI)	Concordance (%)
CYP2C19*2	rs4244285	Splicing defect	28.5 (19.6–37.3)	100
CYP2C19*3	rs4986893	W212X	9.5 (3.7–15.2)	100
CYP2C19*17	rs12248560	−806C>T	1 (0.0–2.95)	100
CYP2B6*4	rs2279343	K262R	7 (2.1–12.0)	100
CYP2B6*6	rs3745274, rs2279343	Q172H, K262R	15.5 (8.4–22.5)	100
CYP2B6*9	rs3745274	Q172H	0.5 (0.0–1.9)	100
CYP3A4*18	rs28371759	L293P	1.5 (0.0–3.89)	100
CYP3A5*3	rs776746	Splicing defect	76.5 (64.2–84.8)	100
MDR1 2677G>A	rs2032582	A893ST	17.5 (10.1–24.9)	100
MDR1 2677G>T	rs2032582	A893ST	38 (28.5–47.5)	100
MDR1 3435 C>T	rs1045642	I1145I	36.5 (27.1–45.9)	100
P2Y12 H2	rs2046934	-	17.5 (10.1–24.9)	100

**Table 4.** Comparative analysis of commonly used genotype platforms.

Assay Name	Assay Type	Cost per Genotype ($) a	Application	Detection Capacity b	Flexibility	Open Source Reference	Reference
SNPlex	OLA/PCR	0.24	~48 SNP	504	−	Protocol No. (cms_042019) c	[27]
HRM	Melting TM	0.3	1 SNP	>13,800	−	HRM protocol d	[28]
Sequenom	Primer Extension	0.2–0.4	40–50 SNP	1536	+	Sequenom protocol e	[29]
SNaPshot (MSSE)	Primer Extension	1	~12 SNP	3840	+	SNaPshot protocol c	[30]
Taqman	5′-nuclease reaction	2.39	1 SNP	>30,000	−	Protocol No. (cms_042998) c	[31]
Pyrosequencing	enzymatic reaction	4–12	~3 SNP	>14,500	−	Pyrosequencing protocol f	[32,33]

OLA: *Oligonucleotide Ligation Assay*, HRM: *High Resolution Melting*.

a Genotyping cost was from the literatures and open source of company;

b The detectable number of samples a day was estimated by the fulltime use of the corresponding machine;

c http://www3.appliedbiosystems.com;

d https://cssportal.roche.com/;

e http://www.sequenom.com/;

f http://www.qub.ac.uk/.
